# The Repression of the HMGB1-TLR4-NF-κB Signaling Pathway by Safflower Yellow May Improve Spinal Cord Injury

**DOI:** 10.3389/fnins.2021.803885

**Published:** 2021-12-24

**Authors:** Lu Wang, Benson O. A. Botchway, Xuehong Liu

**Affiliations:** ^1^Department of Histology and Embryology, Medical College, Shaoxing University, Shaoxing, China; ^2^Institute of Neuroscience, Zhejiang University School of Medicine, Hangzhou, China

**Keywords:** safflower yellow, spinal cord injury, inflammatory reaction, glial scar, the HMGB1-TLR-4-NF-κB signaling pathway

## Abstract

Spinal cord injury (SCI) often results in abnormal sensory and motor functions. Current interventions for SCI in the clinical setting are not effective partly due to the complexity concerning its pathophysiological mechanism. In the wake of SCI, considerable inflammatory cells assemble around the injured area that induces a series of inflammatory reactions and aggravates tissue lesions, thereby affecting the recovery of the damaged nerve tissue. Therefore, the inhibition of inflammatory responses can improve the repair of the injured spinal cord tissue. Safflower Yellow (SY) is the main active ingredient of Carthamus tinctorius. SY has anti-inflammatory effect, as it can inhibit IκBα phosphorylation to impede the NF-κB signaling pathway and p53 nuclear translocation. Besides, SY can limit the release of pro-inflammatory factors, which in turn may alleviate secondary SCI and prevent further complications. In this report, we analyze the pathophysiological mechanism of SCI, the role of inflammatory responses, and how SY interferes with the HMGB1-TLR-4-NF-κB signaling pathway to attenuate inflammatory responses in SCI.

## Introduction

Spinal cord injury (SCI) is a serious central nervous system injury (Ahuja et al., 2017; Hodgetts and Harvey, 2017). In the past decade, significant number of people have suffered from SCI, with its incidence rate still on the rise. According to the National Spinal Cord Injury Statistical Center, there are about 12,500 new SCI cases each year in the North America (Alizadeh et al., 2019). SCI impairs sensorimotor circuits, culminating in motor and sensory dysfunctions (Hilton and Tetzlaff, 2018; Ganzer et al., 2020). SCI considerably affects an individual’s quality of life, and causes an immense social and economic burden (Schattling et al., 2019). To date, the neuron-regenerative repair of SCI continues to be a challenge in the clinical setting (Lindsay et al., 2020). Although several factors could be attributed to this problem, the two main factors concerning the ineffective treatment of SCI are persistent neuro-inflammation and glial scar formation (Yoshizaki et al., 2021). After SCI, astrocytes around the lesion are activated under the action of inflammatory factors. These reactive astrocytes aggregate around the lesion and form glial scars to protect undamaged spinal cord tissue that impedes axonal regeneration (Okada et al., 2018).

Physical trauma can cause the rupture of blood vessels of the spinal cord, damage the blood spinal cord barrier, and result in local bleeding and ischemia, edema, and inflammation, and cell-death (Tran et al., 2018). SCI has two phases: primary SCI and secondary SCI (Hachem et al., 2017). Primary SCI is usually a mechanical damage that causes the destruction of the blood spinal cord barrier and induces local inflammatory responses (Stahel et al., 2012). Secondary injury occurs several hours, days or weeks after the primary SCI. This happens under the action of inflammatory factors, with secondary injury aggravating the damage to the spinal cord tissue (Fan et al., 2013; Tran et al., 2018). Cells within the lesion sites release ATP, DNA, glutamate and free radicals, leading to the formation of a post-damaged cytotoxic environment (Ahuja and Fehlings, 2016). In view of this, inflammatory responses are significant players in secondary SCI (Bethea and Dietrich, 2002).

## The Role of Inflammatory Reaction in SCI Progression

Inflammatory reaction is a protective mechanism of the body. However, excessive and persistent inflammatory microenvironment can hinder spinal cord repair (Li X. et al., 2020). In the wake of SCI, myelin debris are formed, which triggers complement-mediated inflammatory reaction (Kopper and Gensel, 2018). A distinctive consequence of SCI is the upregulation of multiple families of inflammatory molecules that involve cytokines and chemokines (Rice et al., 2007). Inflammatory reaction may aggravate SCI, and cause neuronal cell death, neurodegeneration, and neuroinflammation (Polcyn et al., 2020). Neuroinflammation is one of the key factors that drives secondary SCI (Gaojian et al., 2020). SCI can give rise to a comprehensive inflammatory cascade response induced by the activation of innate immune cells (microglia and astrocyte), leukocytes (neutrophil and macrophage), and neuronal cell death. These cells release pro-inflammatory cytokines, chemokines, free radicals, excitatory toxic amino acids, and nitric oxide (NO) (Hausmann, 2003; Anwar et al., 2016). The pro-inflammatory macrophage and the anti-inflammatory phenotype of the immune cells aggregate at the damage sites to initiate an immune inflammatory response following SCI (Rice et al., 2007; Fan et al., 2020). After SCI, astrocytes play a vital role in SCI pathology through a phenotypic change called reactive cells (Hara et al., 2017). Reactive astrocytes are commonly divided into A1 and A2 types, which are analogous to macrophages M1 and M2 (Liddelow and Barres, 2017; Liddelow et al., 2017; Vismara et al., 2020). Microglia refers to macrophage in the central nervous system (CNS) (Yu et al., 2021). Noteworthy is that invasive macrophages have different functions from microglia (Milich et al., 2019). Classical activated neuro-inflammatory microglia can induce the production of A1 reactive astrocytes (Liddelow et al., 2017). Activated macrophage/microglia are polarized into M1 and M2 sub-types, exhibiting pro-inflammatory and anti-inflammatory effects following SCI (Ransohoff, 2016; Lin et al., 2020). For instance, M1 phenotype generate pro-inflammatory cytokines (such as TNF-α and IL-1β), while M2 phenotype may curtail inflammation via IL-4 and IL-10 cytokines (Jiang et al., 2017). The ratio of M1 to M2 influences the microenvironment of the spinal cord tissue after injury, as the augmentation of M1 phenotype after SCI will negatively affect the injury repair (Fan et al., 2018). Besides, reactive oxygen species (ROS) can lead to cell and tissue dysfunction through the oxidation of DNA and cell membranes, which further causes inflammation (Hervera et al., 2018; Kertmen et al., 2018). SCI comprises of three stages; acute stage, acute secondary stage, and chronic stage (Nukolova et al., 2018).

### The Role of Inflammatory Reaction in Acute Spinal Cord Injury

**FIGURE 1:**
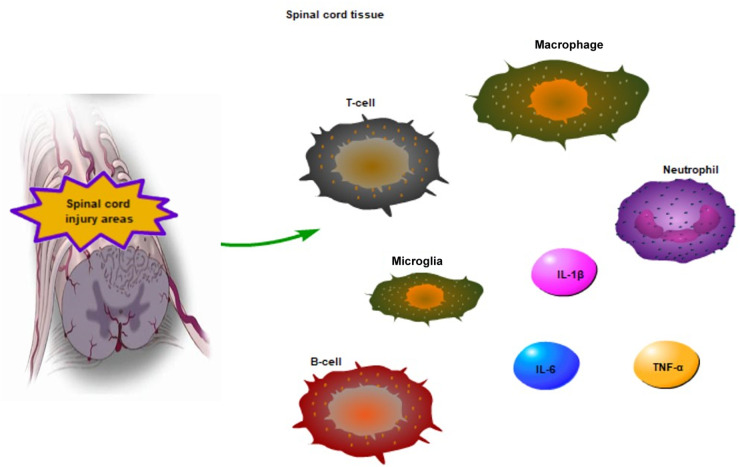
The inflammatory reaction in acute SCI areas. The macrophage, T-cell, neutrophil invade damaged sites following SCI, T and B cells being to gradually increase within 1 week of injury. Moreover, macrophage infiltration and the activation of microglia regulate the release of pro-inflammatory factors, such as TNF-α, IL-6, and IL-1β in acute SCI.

Acute SCI is one of the stages of SCI. At this stage, cell fragments are formed and intracellular proteins are released as potent inflammatory stimuli. These injury-exposed fragment signals, also known as damage-associated molecular patterns (DAMPs), activate pattern recognition receptors (PRRs) on inflammatory cells after SCI (Orr and Gensel, 2018). The acute SCI includes primary and secondary injuries. Oxidative stress leads to the release of cytoplasmic components and mitochondrial dysfunction in primary SCI. Secondary injury begins as early as minutes after the primary SCI, and involves spinal cord ischemia and free radical-mediated peroxidation (Albayar et al., 2019; Pinchi et al., 2019). Oxidative stress is the main cause of neuronal tissue damage, as it can initiate cytotoxicity by enhancing lipid peroxidation in damaged neuronal tissue (Guan et al., 2020). In particular, lipid peroxidation is extremely important in acute SCI (Kwon et al., 2004). Secondary SCI has inflammatory reaction that leads to edema and hemorrhage, which in turn aggravates the injured area. Macrophages, neutrophils and T-cells invade damaged sites, leading to blood-brain barrier disruption (Lambrechts and Cook, 2021; Figure 1). The first infiltrated inflammatory cells are neutrophils, which peak around day 1 after acute SCI. Neutrophils decrease within 1 week of injury, while monocytes increase in the spinal cord. Similarly, T and B-lymphocytes being to gradually increase during the first week after injury (Wu et al., 2019; Figure 1). Subsequent to acute SCI, ischemia leads to the formation of an acidic environment. Moreover, macrophage infiltration and the activation of microglia further promote the release of pro-inflammatory factors, including TNF-α, IL-1β, and interleukin 6 (IL-6) (Xi et al., 2021; Figure 1). The microglia are the key immune cell type in CNS (DiSabato et al., 2016). Under normal circumstances, microglia perform immune defense mechanisms, regulate neuronal and synaptic activities, secrete nutritional factors and support neuronal survival and axon growth in CNS (DiSabato et al., 2016; Gaudet and Fonken, 2018). The microglia can be strongly activated and carry out double-edged tasks following SCI (Gaudet and Fonken, 2018). The microglial and macrophages can have beneficial roles in acute SCI. A large number of macrophages and microglia are recruited in the lesion epicenter within 7 days after SCI (Stirling and Yong, 2008). Activated microglial and macrophages secrete products that promote axon growth. Zymosan-activated macrophages create a growth-microenvironment to increase the density of axons *in vivo* (Gensel et al., 2009). A study has showed that M1 phenotype cells can produce proteases and oxidative metabolities to kill neurons and glia, conversely, M2 phenotype cells can contribute to tissue repair via downregulating inflammatory responses in SCI (Kigerl et al., 2009). Acute SCI leads to chronic SCI, and chronic complications after acute SCI are detrimental (Chen et al., 2020).

### The Role of Inflammatory Reaction in Chronic Spinal Cord Injury

**FIGURE 2:**
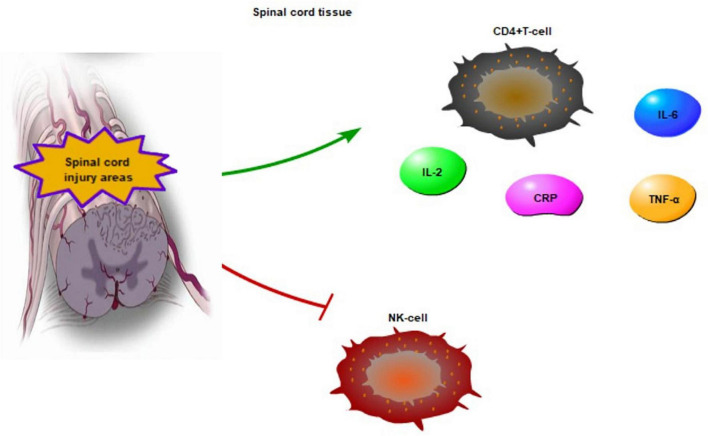
The inflammatory reaction in chronic SCI areas. CD4 + T-cell, CRP, IL-6 are increased, IL-2 and TNF-α are activated to enhance the NF-κB transcriptional activity in chronic SCI. Meanwhile, the activity of NK cell is inhibited.

Systemic inflammation is key to chronic SCI (Diaz et al., 2021). Systemic inflammatory markers, such as c-reactive protein (CRP) and IL-6, are increased after chronic SCI (Hart et al., 2016; Lynch et al., 2017; Dugan et al., 2021). Chronic SCI can intensify IL-2 and TNF-α levels to upregulate the NF-κB transcriptional activity (Yarar-Fisher et al., 2016). Microglia appears to be strongly related to chronic neuroinflammation after SCI, and microglial cells expressing TNF-α may transform the polarization of astrocytes to neurotoxic phenotypes (Yoshizaki et al., 2021). Besides, natural killer cell numbers, cytotoxic activity levels, and T-lymphocytes in patients with chronic SCI exhibit abnormal function (Figure 2). There are indications that CD4^+^ T cells are increased in the spinal tissue (Monahan et al., 2015; Herman et al., 2018). Therefore, several factors such as IL-2, IL-6, CRP, TNF-α and CD4^+^T cells can be activated, however, NK cells can be inhibited in chronic SCI (Figure 2). Chronic SCI is a period of stabilization and low activity, where the nerve function around the injured areas gradually decreases (Rodríguez-Barrera et al., 2017).

## Safflower Yellow Can Inhibit Inflammatory Reaction

### The Biological Role of Safflower Yellow

Carthamus tinctorius is a plant of Compositae or Asteraceae family (Delshad et al., 2018). Safflower is the dry flower of Cathamus tinctorius, a commonly used traditional Chinese medicine that has been reported to improve trauma, gynecological disease, cardiovascular conditions, blood circulation, and remove blood stasis (Wang et al., 2011). SY is the effective component of safflower water-soluble extract, with its main component being hydroxysafflower yellow (Asgarpanah and Kazemivash, 2013; Li H. et al., 2020; Wang et al., 2020). The molecular formula of SY is C60H74O38, contains hydroxyl groups, carbonyl groups, aromatic rings and conjugated carbonyl groups. Hydroxysafflor yellow A-4′-O-b-D-glucopyranoside and 3′-hydroxyhydroxysafflor yellow A are separated from the SY (Zhang et al., 2020). SY has anti-infection and anti-inflammatory properties, and has been used for the clinical treatment of patients who suffer from severe sepsis and septic shock (Li et al., 2016). Furthermore, SY has anti-fibrotic (Wang et al., 2011), anti-oxidative (Wang et al., 2020), anti-coagulative (Sun et al., 2010), anti-obesity (Yan et al., 2020), anti-calcium-antagonist (Du et al., 2019), and neuroprotective effects (Pang et al., 2020). In recent times, the mediation of SY in inflammation has attracted significant attention.

### Safflower Yellow Inhibits the HMGB1-TLR4-NF-κB Signaling Pathway

**FIGURE 3:**
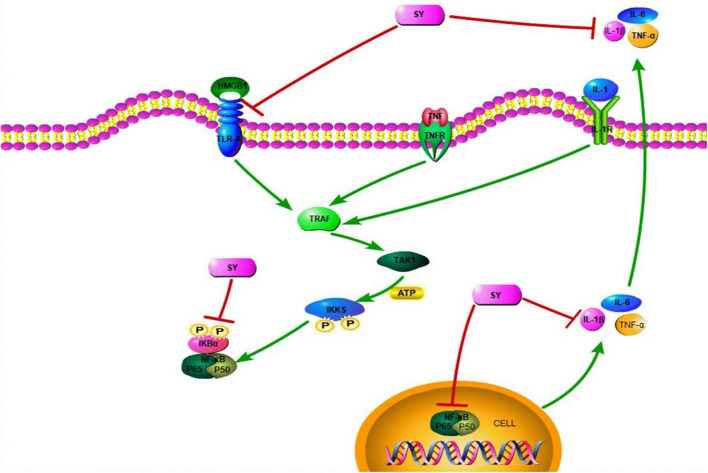
The role of SY in SCI. SY moderates the release of pro-inflammatory factors (TNF-α, IL-6, and IL-1β). Additionally, SY inhibits IκBα phosphorylation and p53 nuclear translocation. Thus, SY can suppress the TLR-4-NF-κB signaling pathway.

High mobility group box 1 protein (HMGB1) is a nuclear non-histone DNA-binding protein expressed in all nuclear animal cells, and can be used as a potent inflammatory late mediator when passively secreted during inflammatory response (Scaffidi et al., 2010; Li et al., 2015). HMGB1, which can stimulate neuroinflammatory responses under deleterious conditions, is a damage-associated molecular pattern (DAMP) molecule (Yu et al., 2019). HMGB1 can induce intracellular signaling pathway by interacting with at least three pattern recognition receptors: Toll-like receptor-2 (TLR-2) and TLR-4, and the receptor for advanced glycation products (PAGE) (van Zoelen et al., 2009). Among them, TLR-2 and TLR-4 are key players, while PAGE has a minimal role (Park et al., 2004). The migration ability of breast cancer cells is closely related to HMGB1 (Lv et al., 2016). HMGB1 can be released from the nucleus to the cytoplasm under damage conditions to activate TLR4 signaling pathway and play a biological role (Lv et al., 2016; Antón et al., 2017; Xu et al., 2020). HMGB1 and TLR-4 interactions may lead to NF-κB upregulation, which results in producing and releasing inflammatory cytokines, such as IL-1β, TNF-α and IL-6 (Zhang et al., 2007; Kang et al., 2015; Wang et al., 2015; Xu et al., 2020; Figure 3). The HMGB1-TLR4-NF-κB signaling pathway is an inflammatory signaling pathway that mediates multiple inflammation-related pathways (Sun et al., 2019). NF-κB is a well-established inflammatory transcription factors produced by almost all animal cells. More importantly, the NF-κB signaling pathway has a significant number of target genes that can regulate a variety of biological functions, including inflammation, apoptosis, cell adhesion, cell stress response, and immunity (Jing and Lee, 2014). More importantly, the NF-κB signaling pathway is instrumental in inflammation (Ma and Hottiger, 2016). There is a positive feedback mechanism between inflammation and the NF-κB signaling pathway after SCI (Karova et al., 2019). Exposing neutrophils or macrophages to HMGB1 can lead to enhanced NF-κB signaling pathway and pro-inflammatory cytokine expression (Park et al., 2004). Interestingly, SY can improve inflammatory response and exert effect on inflammatory factors, like TNF-α, IL-1(IL-1β), and IL-6 (Zhou et al., 2018; Du et al., 2019; Figure 3). Furthermore, SY can inhibit the activation of the NF-κB signaling pathway by suppressing IκBα phosphorylation and cell nucleus translocation of p65 (Li et al., 2013; Figure 3). Moreover, SY may suppress the NF-κB signaling pathway by restricting the TNF-α (Wang et al., 2020). SY has a significant role in the minimization of ROS level (Lu et al., 2019). Also, SY can downregulate the TLR-4 expression (Yang et al., 2015; Figure 3). Besides, SY can transform microglia from inflammatory M1 to anti-inflammatory M2, which then plays an anti-inflammatory role by hindering the TLR-4-NF-κB signaling pathway (Yang et al., 2016). SY has been widely studied in various diseases, most especially, SCI (Table 1).

**TABLE 1:** Beneficial effects of SY in diseases.

Diseases	Species	Doses	Outcome	References
Pulmonary fibrosis	Rats	0.25 mg/ml	SY can inhibit α-SMA mRNA expression in lung fibroblast.	Wang et al., 2011
Osteoarthritis	Rats	50 μg/ml	SY can regulate NF-κB/SIRT1/AMPK signaling pathway, and prevent inflammation.	Wang et al., 2020
Focal cerebral	Rats	8 mg/kg	HSYA suppresses thrombin formation and inflammatory responses.	Sun et al., 2010
Obesity	Mice	120 mg/kg	SY may improve insulin sensitivity.	Yan et al., 2020
Cerebral ischemia	Rats	8 mg/kg	SYB can activate AMPK and reduce NF-κB mediated inflammation.	Du et al., 2019
Alzheimer’s disease (AD)	Mice	30 mg/kg	SY can improve learning and memory functions.	Pang et al., 2020
CNS	Cell	80 μg/ml	SY can inhibit inflammatory response.	Yang et al., 2016
AD	Mice	100 mg/kg	SY can improve AD by decreasing the expression of proteins related to β-amyloid formation.	Shi et al., 2018
ROS	Cell	/	SYB can effectively reduce ROS generation by decreasing NADPH oxidase activity.	Wang et al., 2013
Bone fracture	Cell	18 μg/ml	SY can promote angiogenesis to improve bone fracture.	Tang et al., 2018
Obesity/diabetic	Mice	80 μg/ml	SY can reduce body fat mass and improve insulin sensitivity.	Zhu et al., 2016
Cardiovascular disease (CVD)	Mice	25 μg/ml	SY has an effect on angiotensin II-induced adventitial fibroblast proliferation.	Liu et al., 2014
CVD	Cell	20 μg/ml	HSYA can decrease PDGF-BB-induced proliferation, migration, and Akt signaling pathway.	Song et al., 2014
AD	Cell	10 μg/ml	HSYA can inhibit neuroinflammation by reducing Aβ1-42-induced cytotoxicity in BV-2 cells.	Zhang et al., 2014
SCI	Rabbits	90 μg/ml	SY can improve SCI by enhancing Bcl-2 expression and inhibiting Bax and caspase-3 activation.	Zhou et al., 2013

## Concluding Remarks

Inflammation plays an important role in SCI, which have been expounded in this report. The NF-κB is a central transcription factor of inflammatory mediators, and the neuroinflammatory response caused by activated microglia through the NF-κB pathway is a consequential contributing factor to secondary injury (Chen et al., 2018). The HMGB1-TLR-4-NF-κB signaling is an inflammatory pathway upregulated during SCI. Particularly, HMGB1 and TLR-4 interactions can lead to NF-κB upregulation, which in turn results in the formation and release of inflammatory cytokines at increasing levels in secondary SCI (Zhang et al., 2007; Wang et al., 2015). SY has several pharmacological effects, such as anti-inflammation and anti-oxidation. SY may mitigate the release of pro-inflammatory factors, TNF-α, IL-1β, and IL-6. Far more, SY can inhibit the HMGB1-TLR-4-NF-κB signaling pathway to ameliorate inflammatory response and offer protection to the spinal cord in the event of an injury. Notwithstanding, the specific molecular mechanism of HMGB-TLR-4-NF-κB following SCI are presently unclear, and warrants further thorough investigations using appropriate experimental models.

## Author Contributions

XL designed the study. LW, BOAB, and XL prepared the first draft of the manuscript and revised the manuscript. All authors approved the final manuscript.

## Conflict of Interest

The authors declare that the research was conducted in the absence of any commercial or financial relationships that could be construed as a potential conflict of interest.

## Publisher’s Note

All claims expressed in this article are solely those of the authors and do not necessarily represent those of their affiliated organizations, or those of the publisher, the editors and the reviewers. Any product that may be evaluated in this article, or claim that may be made by its manufacturer, is not guaranteed or endorsed by the publisher.

## References

[B1] Ahuja C. S. Fehlings M. (2016). concise review: bridging the gap: novel neuroregenerative and neuroprotective strategies in spinal cord injury. *Stem Cells Transl. Med.* 5 914–924. 10.5966/sctm.2015-0381 27130222 PMC4922857

[B2] Ahuja C. S. Nori S. Tetreault L. Wilson J. Kwon B. Harrop J. (2017). Traumatic spinal cord injury-repair and regeneration. *Neurosurgery* 80 S9–S22. 10.1093/neuros/nyw080 28350947

[B3] Albayar A. A. Roche A. Swiatkowski P. Antar S. Ouda N. Emara E. (2019). Biomarkers in spinal cord injury: prognostic insights and future potentials. *Front. Neurol.* 10:27. 10.3389/fneur.2019.00027 30761068 PMC6361789

[B4] Alizadeh A. Dyck S. M. Karimi-Abdolrezaee S. (2019). Traumatic spinal cord injury: an overview of pathophysiology, models and acute injury mechanisms. *Front. Neurol.* 10:282. 10.3389/fneur.2019.00282 30967837 PMC6439316

[B5] Antón M. Alén F. Gómez de Heras R. Serrano A. Pavón F. J. Leza J. C. (2017). Oleoylethanolamide prevents neuroimmune HMGB1/TLR4/NF-kB danger signaling in rat frontal cortex and depressive-like behavior induced by ethanol binge administration. *Addict. Biol.* 22 724–741. 10.1111/adb.12365 26857094

[B6] Anwar M. A. Al Shehabi T. S. Eid A. H. (2016). Inflammogenesis of secondary spinal cord injury. *Front. Cell Neurosci.* 10:98. 10.3389/fncel.2016.00098 27147970 PMC4829593

[B7] Asgarpanah J. Kazemivash N. (2013). Phytochemistry, pharmacology and medicinal properties of *Carthamus tinctorius* L. *Chin. J. Integr. Med.* 19 153–159. 10.1007/s11655-013-1354-5 23371463

[B8] Bethea J. R. Dietrich W. D. (2002). Targeting the host inflammatory response in traumatic spinal cord injury. *Curr. Opin. Neurol.* 15 355–360. 10.1097/00019052-200206000-00021 12045737

[B9] Chen G. Zhou Z. Sha W. Wang L. Yan F. Yang X. (2020). A novel CX3CR1 inhibitor AZD8797 facilitates early recovery of rat acute spinal cord injury by inhibiting inflammation and apoptosis. *Int. J. Mol. Med.* 45 1373–1384. 10.3892/ijmm.2020.4509 32323731 PMC7138267

[B10] Chen S. Ye J. Chen X. Shi J. Wu W. Lin W. (2018). Valproic acid attenuates traumatic spinal cord injury-induced inflammation via STAT1 and NF-κB pathway dependent of HDAC3. *J. Neuroinflammation* 15:150. 10.1186/s12974-018-1193-6 29776446 PMC5960086

[B11] Delshad E. Yousefi M. Sasannezhad P. Rakhshandeh H. Ayati Z. (2018). Medical uses of *Carthamus tinctorius* L. (Safflower): a comprehensive review from traditional medicine to modern medicine. *Electron. Phys.* 10 6672–6681. 10.19082/6672 29881530 PMC5984022

[B12] Diaz D. Lopez-Dolado E. Haro S. Monserrat J. Martinez-Alonso C. Balomeros D. (2021). Systemic inflammation and the breakdown of intestinal homeostasis are key events in chronic spinal cord injury patients. *Int. J. Mol. Sci.* 22:744. 10.3390/ijms22020744 33451043 PMC7828526

[B13] DiSabato D. J. Quan N. Godbout J. P. (2016). Neuroinflammation: the devil is in the details. *J. Neurochem.* 139 Suppl 2(Suppl. 2) 136–153. 10.1111/jnc.13607 26990767 PMC5025335

[B14] Du S. Deng Y. Yuan H. Sun Y. (2019). Safflower yellow B protects brain against cerebral ischemia reperfusion injury through AMPK/NF-kB pathway. *Evid. Based Compl. Alternat. Med.* 2019:7219740. 10.1155/2019/7219740 30854014 PMC6378026

[B15] Dugan E. A. Schachner B. Jergova S. Sagen J. (2021). Intensive locomotor training provides sustained alleviation of chronic spinal cord injury-associated neuropathic pain: a two-year pre-clinical study. *J. Neurotrauma* 38 789–802. 10.1089/neu.2020.7378 33218293

[B16] Fan B. Wei Z. Yao X. Shi G. Cheng X. Zhou X. (2018). Microenvironment imbalance of spinal cord injury. *Cell Transplant.* 27 853–866. 10.1177/0963689718755778 29871522 PMC6050904

[B17] Fan H. Liu X. Tang H. B. Xiao P. Wang Y. Z. Ju G. (2013). Protective effects of batroxobin on spinal cord injury in rats. *Neurosci. Bull.* 29 501–508. 10.1007/s12264-013-1354-7 23852558 PMC5561936

[B18] Fan H. Tang H. B. Chen Z. Wang H. Q. Zhang L. Jiang Y. (2020). Inhibiting HMGB1-RAGE axis prevents pro-inflammatory macrophages/microglia polarization and affords neuroprotection after spinal cord injury. *J. Neuroinflammation* 17:295. 10.1186/s12974-020-01973-4 33036632 PMC7547440

[B19] Ganzer P. D. Colachis S. C. IV Schwemmer M. A. Friedenberg D. A. Dunlap C. F. Swiftney C. E. (2020). Restoring the sense of touch using a sensorimotor demultiplexing neural interface. *Cell* 181 763–773.e12. 10.1016/j.cell.2020.03.054 32330415

[B20] Gaojian T. Dingfei Q. Linwei L. Xiaowei W. Zheng Z. Wei L. (2020). Parthenolide promotes the repair of spinal cord injury by modulating M1/M2 polarization via the NF-κB and STAT 1/3 signaling pathway. *Cell Death Discov.* 6:97. 10.1038/s41420-020-00333-8 PMC7538575 33083018

[B21] Gaudet A. D. Fonken L. K. (2018). Glial cells shape pathology and repair after spinal cord injury. *Neurotherapeutics* 15 554–577. 10.1007/s13311-018-0630-7 29728852 PMC6095774

[B22] Gensel J. C. Nakamura S. Guan Z. van Rooijen N. Ankeny D. P. Popovich P. G. (2009). Macrophages promote axon regeneration with concurrent neurotoxicity. *J. Neurosci.* 29 3956–3968. 10.1523/JNEUROSCI.3992-08.2009 19321792 PMC2693768

[B23] Guan B. Chen R. Zhong M. Liu N. Chen Q. (2020). Protective effect of oxymatrine against acute spinal cord injury in rats via modulating oxidative stress, inflammation and apoptosis. *Metab. Brain Dis.* 35 149–157. 10.1007/s11011-019-00528-8 31840202

[B24] Hachem L. D. Ahuja C. S. Fehlings M. G. (2017). Assessment and management of acute spinal cord injury: from point of injury to rehabilitation. *J. Spinal Cord Med.* 40 665–675. 10.1080/10790268.2017.1329076 28571527 PMC5778930

[B25] Hara M. Kobayakawa K. Ohkawa Y. Kumamaru H. Yokota K. Saito T. (2017). Interaction of reactive astrocytes with type I collagen induces astrocytic scar formation through the integrin-N-cadherin pathway after spinal cord injury. *Nat. Med.* 23 818–828. 10.1038/nm.4354 28628111

[B26] Hart J. E. Morse L. Tun C. G. Brown R. Garshick E. (2016). Cross-sectional associations of pulmonary function with systemic inflammation and oxidative stress in individuals with chronic spinal cord injury. *J. Spinal Cord Med.* 39 344–352. 10.1179/2045772315Y.0000000045 26180939 PMC5073753

[B27] Hausmann O. N. (2003). Post-traumatic inflammation following spinal cord injury. *Spinal Cord* 41 369–378. 10.1038/sj.sc.3101483 12815368

[B28] Herman P. Stein A. Gibbs K. Korsunsky I. Gregersen P. Bloom O. (2018). Persons with chronic spinal cord injury have decreased natural killer cell and increased toll-like receptor/inflammatory gene expression. *J. Neurotrauma* 35 1819–1829. 10.1089/neu.2017.5519 29310515 PMC6033303

[B29] Hervera A. De Virgiliis F. Palmisano I. Zhou L. Tantardini E. Kong G. (2018). Reactive oxygen species regulate axonal regeneration through the release of exosomal NADPH oxidase 2 complexes into injured axons. *Nat. Cell Biol.* 20 307–319. 10.1038/s41556-018-0039-x 29434374

[B30] Hilton B. J. Tetzlaff W. (2018). A brainstem bypass for spinal cord injury. *Nat. Neurosci.* 21 457–458. 10.1038/s41593-018-0099-z 29556026

[B31] Hodgetts S. I. Harvey A. R. (2017). Neurotrophic Factors used to treat spinal cord injury. *Vitam. Horm.* 104 405–457. 10.1016/bs.vh.2016.11.007 28215303

[B32] Jiang J. Luo Y. Qin W. Ma H. Li Q. Zhan J. (2017). Electroacupuncture suppresses the NF-κB signaling pathway by upregulating cylindromatosis to alleviate inflammatory injury in cerebral ischemia/reperfusion rats. *Front. Mol. Neurosci.* 10:363. 10.3389/fnmol.2017.00363 29163038 PMC5681846

[B33] Jing H. Lee S. (2014). NF-κB in cellular senescence and cancer treatment. *Mol. Cells* 37 189–195. 10.14348/molcells.2014.2353 24608805 PMC3969038

[B34] Kang N. Hai Y. Yang J. Liang F. Gao C. J. (2015). Hyperbaric oxygen intervention reduces secondary spinal cord injury in rats via regulation of HMGB1/TLR4/NF-κB signaling pathway. *Int. J. Clin. Exp. Pathol.* 8 1141–1153. 25973000 PMC4396250

[B35] Karova K. Wainwright J. V. Machova-Urdzikova L. Pisal R. V. Schmidt M. Jendelova P. (2019). Transplantation of neural precursors generated from spinal progenitor cells reduces inflammation in spinal cord injury via NF-κB pathway inhibition. *J Neuroinflammation.* 16 12. 10.1186/s12974-019-1394-7 30654804 PMC6335809

[B36] Kertmen H. Celikoglu E. Ozturk O. C. Gürer B. Bozkurt H. Kanat M. A. (2018). Comparative effects of methylprednisolone and tetracosactide (ACTH1-24) on ischemia/reperfusion injury of the rabbit spinal cord. *Arch. Med. Sci.* 14 1459–1470. 10.5114/aoms.2017.65650 30393502 PMC6209702

[B37] Kigerl K. A. Gensel J. C. Ankeny D. P. Alexander J. K. Donnelly D. J. Popovich P. G. (2009). Identification of two distinct macrophage subsets with divergent effects causing either neurotoxicity or regeneration in the injured mouse spinal cord. *J. Neurosci.* 29 13435–13444. 10.1523/JNEUROSCI.3257-09.2009 19864556 PMC2788152

[B38] Kopper T. J. Gensel J. C. (2018). Myelin as an inflammatory mediator: myelin interactions with complement, macrophages, and microglia in spinal cord injury. *J. Neurosci. Res.* 96 969–977. 10.1002/jnr.24114 28696010 PMC5764830

[B39] Kwon B. K. Tetzlaff W. Grauer J. N. Beiner J. Vaccaro A. R. (2004). Pathophysiology and pharmacologic treatment of acute spinal cord injury. *Spine J.* 4 451–464. 10.1016/j.spinee.2003.07.007 15246307

[B40] Lambrechts M. J. Cook J. L. (2021). Nonsteroidal anti-inflammatory drugs and their neuroprotective role after an acute spinal cord injury: a systematic review of animal models. *Glob. Spine J.* 11 365–377. 10.1177/2192568220901689 32875860 PMC8013945

[B41] Li H. Kan B. Song L. Liu Y. Jian X. (2020). Role of the Hippo signaling pathway in safflower yellow pigment treatment of paraquat-induced pulmonary fibrosis. *J. Int. Med. Res.* 48:300060520905425. 10.1177/0300060520905425 32940100 PMC7503030

[B42] Li J. Zhang S. Lu M. Chen Z. Chen C. Han L. (2013). Hydroxysafflor yellow a suppresses inflammatory responses of BV2 microglia after oxygen-glucose deprivation. *Neurosci. Lett.* 535 51–56. 10.1016/j.neulet.2012.12.056 23333598

[B43] Li L. Ling Y. Huang M. Yin T. Gou S. M. Zhan N. Y. (2015). Heparin inhibits the inflammatory response induced by LPS and HMGB1 by blocking the binding of HMGB1 to the surface of macrophages. *Cytokine* 72 36–42. 10.1016/j.cyto.2014.12.010 25562836

[B44] Li X. Yu Z. Zong W. Chen P. Li J. Wang M. (2020). Deficiency of the microglial Hv1 proton channel attenuates neuronal pyroptosis and inhibits inflammatory reaction after spinal cord injury. *J. Neuroinflammation* 17:263. 10.1186/s12974-020-01942-x 32891159 PMC7487532

[B45] Li X. J. Wang R. R. Kang Y. Liu J. Zuo Y. X. Zeng X. F. (2016). Effects of safflower yellow on the treatment of severe sepsis and septic shock: a randomized controlled clinical trial. *Evid Based Compl. Alternat. Med.* 2016:3948795. 10.1155/2016/3948795 26989426 PMC4775808

[B46] Liddelow S. A. Barres B. A. (2017). Reactive astrocytes: production, function, and therapeutic potential. *Immunity* 46 957–967. 10.1016/j.immuni.2017.06.006 28636962

[B47] Liddelow S. A. Guttenplan K. A. Clarke L. E. Bennett F. C. Bohlen C. J. Schirmer L. (2017). Neurotoxic reactive astrocytes are induced by activated microglia. *Nature* 541 481–487. 10.1038/nature21029 28099414 PMC5404890

[B48] Lin J. Huang Z. Liu J. Huang Z. Liu Y. Liu Q. (2020). Neuroprotective effect of ketone metabolism on inhibiting inflammatory response by regulating macrophage polarization after acute cervical spinal cord injury in rats. *Front. Neurosci.* 14:583611. 10.3389/fnins.2020.583611 33192269 PMC7645058

[B49] Lindsay S. L. McCanney G. A. Willison A. G. Barnett S. C. (2020). Multi-target approaches to CNS repair: olfactory mucosa-derived cells and heparan sulfates. *Nat. Rev. Neurol.* 16 229–240. 10.1038/s41582-020-0311-0 32099190

[B50] Liu Y. Tian X. Cui M. Zhao S. (2014). Safflower yellow inhibits angiotensin II-induced adventitial fibroblast proliferation and migration. *J. Pharmacol. Sci.* 126 107–114. 10.1254/jphs.14055fp 25231558

[B51] Lu Q. Y. Ma J. Q. Duan Y. Y. Sun Y. Yu S. Li B. (2019). Carthamin yellow protects the heart against ischemia/reperfusion injury with reduced reactive oxygen species release and inflammatory response. *J. Cardiovasc. Pharmacol.* 74 228–234. 10.1097/FJC.0000000000000710 31356540

[B52] Lv W. Chen N. Lin Y. Ma H. Ruan Y. Li Z. (2016). Macrophage migration inhibitory factor promotes breast cancer metastasis via activation of HMGB1/TLR4/NF kappa B axis. *Cancer Lett.* 375 245–255. 10.1016/j.canlet.2016.02.005 26952810

[B53] Lynch M. Duffell L. Sandhu M. Srivatsan S. Deatsch K. Kessler A. (2017). Effect of acute intermittent hypoxia on motor function in individuals with chronic spinal cord injury following ibuprofen pretreatment: a pilot study. *J. Spinal Cord Med.* 40 295–303. 10.1080/10790268.2016.1142137 26856344 PMC5472017

[B54] Ma B. Hottiger M. O. (2016). Crosstalk between Wnt/β-Catenin and NF-κB signaling pathway during Inflammation. *Front. Immunol.* 7:378. 10.3389/fimmu.2016.00378 27713747 PMC5031610

[B55] Milich L. M. Ryan C. B. Lee J. K. (2019). The origin, fate, and contribution of macrophages to spinal cord injury pathology. *Acta Neuropathol.* 137 785–797. 10.1007/s00401-019-01992-3 30929040 PMC6510275

[B56] Monahan R. Stein A. Gibbs K. Bank M. Bloom O. (2015). Circulating T cell subsets are altered in individuals with chronic spinal cord injury. *Immunol. Res.* 63 3–10. 10.1007/s12026-015-8698-1 26440591 PMC4648984

[B57] Nukolova N. V. Aleksashkin A. D. Abakumova T. O. Morozova A. Y. Gubskiy I. L. Kirzhanova EA (2018). Multilayer polyion complex nanoformulations of superoxide dismutase 1 for acute spinal cord injury. *J. Control Release* 270 226–236. 10.1016/j.jconrel.2017.11.044 29196042

[B58] Okada S. Hara M. Kobayakawa K. Matsumoto Y. Nakashima Y. (2018). Astrocyte reactivity and astrogliosis after spinal cord injury. *Neurosci. Res.* 126 39–43. 10.1016/j.neures.2017.10.004 29054466

[B59] Orr M. B. Gensel J. C. (2018). Spinal cord injury scarring and inflammation: therapies targeting glial and inflammatory responses. *Neurotherapeutics* 15 541–553. 10.1007/s13311-018-0631-6 29717413 PMC6095779

[B60] Pang J. Hou J. Zhou Z. Ren M. Mo Y. Yang G. (2020). Safflower yellow improves synaptic plasticity in APP/PS1 mice by regulating microglia activation phenotypes and BDNF/TrkB/ERK signaling pathway. *Neuromol. Med.* 22 341–358. 10.1007/s12017-020-08591-6 32048142

[B61] Park J. S. Svetkauskaite D. He Q. Kim J. Y. Strassheim D. Ishizaka A. (2004). Involvement of toll-like receptors 2 and 4 in cellular activation by high mobility group box 1 protein. *J. Biol. Chem.* 279 7370–7377. 10.1074/jbc.M306793200 14660645

[B62] Pinchi E. Frati A. Cantatore S. D’Errico S. Russa R. Maiese A. (2019). Acute spinal cord injury: a systematic review investigating miRNA families involved. *Int. J. Mol. Sci.* 20:1841. 10.3390/ijms20081841 31013946 PMC6515063

[B63] Polcyn R. Capone M. Matzelle D. Hossain A. Chandran R. Banik N. L. (2020). Enolase inhibition alters metabolic hormones and inflammatory factors to promote neuroprotection in spinal cord injury. *Neurochem. Int.* 139:104788. 10.1016/j.neuint.2020.104788 32650031 PMC7483987

[B64] Ransohoff R. M. (2016). A polarizing question: do M1 and M2 microglia exist? *Nat. Neurosci.* 19 987–991. 10.1038/nn.4338 27459405

[B65] Rice T. Larsen J. Rivest S. Yong V. W. (2007). Characterization of the early neuroinflammation after spinal cord injury in mice. *J. Neuropathol. Exp. Neurol.* 66 184–195. 10.1097/01.jnen.0000248552.07338.7f 17356380

[B66] Rodríguez-Barrera R. Flores-Romero A. Fernández-Presas A. M. García-Vences E. Silva-García R. Konigsberg M. (2017). Immunization with neural derived peptides plus scar removal induces a permissive microenvironment, and improves locomotor recovery after chronic spinal cord injury. *BMC Neurosci.* 18:7. 10.1186/s12868-016-0331-2 28056790 PMC5217189

[B67] Scaffidi P. Misteli T. Bianchi M. E. (2010). Release of chromatin protein HMGB1 by necrotic cells triggers inflammation. Nature. 2002; 418(6894):191-5. *Erratum Nat.* 467:622. 10.1038/nature00858 12110890

[B68] Schattling B. Engler J. B. Volkmann C. Rothammer N. Woo M. S. Petersen M. (2019). Bassoon proteinopathy drives neurodegeneration in multiple sclerosis. *Nat. Neurosci.* 22 887–896. 10.1038/s41593-019-0385-4 31011226

[B69] Shi X. M. Zhang H. Zhou Z. J. Ruan Y. Y. Pang J. Zhang L. (2018). Effects of safflower yellow on beta-amyloid deposition and activation of astrocytes in the brain of APP/PS1 transgenic mice. *Biomed. Pharmacother.* 98 553–565. 10.1016/j.biopha.2017.12.099 29288971

[B70] Song Y. Long L. Zhang N. Liu Y. (2014). Inhibitory effects of hydroxysafflor yellow A on PDGF-BB-induced proliferation and migration of vascular smooth muscle cells via mediating Akt signaling. *Mol. Med. Rep.* 10 1555–1560. 10.3892/mmr.2014.2336 24939805

[B71] Stahel P. F. VanderHeiden T. Finn M. A. (2012). Management strategies for acute spinal cord injury: current options and future perspectives. *Curr. Opin. Crit. Care* 18 651–660. 10.1097/MCC.0b013e32835a0e54 23104069

[B72] Stirling D. P. Yong V. W. (2008). Dynamics of the inflammatory response after murine spinal cord injury revealed by flow cytometry. *J. Neurosci. Res.* 86 1944–1958. 10.1002/jnr.21659 18438914

[B73] Sun L. Zhao L. Li P. Liu X. Liang F. Jiang Y. (2019). Effect of hyperbaric oxygen therapy on HMGB1/NF-κB expression and prognosis of acute spinal cord injury: a randomized clinical trial. *Neurosci. Lett.* 692 47–52. 10.1016/j.neulet.2018.10.059 30391318

[B74] Sun X. Wei X. Qu S. Zhao Y. Zhang X. (2010). Hydroxysafflor yellow a suppresses thrombin generation and inflammatory responses following focal cerebral ischemia-reperfusion in rats. *Bioorg. Med. Chem. Lett.* 20 4120–4124. 10.1016/j.bmcl.2010.05.076 20542424

[B75] Tang Z. Xie H. Jiang S. Cao S. Pu Y. Zhou B. (2018). Safflower yellow promotes angiogenesis through p-VHL/HIF-1α/VEGF signaling pathway in the process of osteogenic differentiation. *Biomed. Pharmacother.* 107 1736–1743. 10.1016/j.biopha.2018.06.119 30257392

[B76] Tran A. P. Warren P. M. Silver J. (2018). The biology of regeneration failure and success after spinal cord injury. *Physiol. Rev.* 98 881–917. 10.1152/physrev.00017.2017 29513146 PMC5966716

[B77] van Zoelen M. A. Yang H. Florquin S. Meijers J. C. Akira S. Arnold B. (2009). Role of toll-like receptors 2 and 4, and the receptor for advanced glycation end products in high-mobility group box 1-induced inflammation in vivo. *Shock* 31 280–284. 10.1097/SHK.0b013e318186262d 19218854 PMC4535325

[B78] Vismara I. Papa S. Veneruso V. Mauri E. Mariani A. De Paola M. (2020). Selective modulation of A1 Astrocytes by drug-loaded nano-structured gel in spinal cord injury. *ACS Nano* 14 360–371. 10.1021/acsnano.9b05579 31887011

[B79] Wang C. Gao Y. Zhang Z. Chi Q. Liu Y. Yang L. (2020). Safflower yellow alleviates osteoarthritis and prevents inflammation by inhibiting PGE2 release and regulating NF-κB/SIRT1/AMPK signaling pathways. *Phytomedicine* 78:153305. 10.1016/j.phymed.2020.153305 32871523

[B80] Wang C. He Y. Yang M. Sun H. Zhang S. Wang C. (2013). Safflor yellow B suppresses angiotensin II-mediated human umbilical vein cell injury via regulation of Bcl-2/p22(phox) expression. *Toxicol. Appl. Pharmacol.* 273 59–67. 10.1016/j.taap.2013.08.018 23994555

[B81] Wang L. Jin M. Zang B. X. Wu Y. (2011). Inhibitory effect of safflor yellow on pulmonary fibrosis. *Biol. Pharm. Bull.* 34 511–516. 10.1248/bpb.34.511 21467638

[B82] Wang Y. S. Li Y. Y. Wang L. H. Kang Y. Zhang J. Liu Z. Q. (2015). Tanshinone IIA attenuates chronic pancreatitis-induced pain in rats via downregulation of HMGB1 and TRL4 expression in the spinal cord. *Pain Phys.* 18 E615–E628. 26218952

[B83] Wu F. Ding X. Y. Li X. H. Gong M. J. An J. Q. Lai J. H. (2019). Cellular inflammatory response of the spleen after acute spinal cord injury in rat. *Inflammation* 42 1630–1640. 10.1007/s10753-019-01024-y 31102125

[B84] Xi K. Gu Y. Tang J. Chen H. Xu Y. Wu L. (2021). Microenvironment-responsive immunoregulatory electrospun fibers for promoting nerve function recovery. Nat Commun. 2020; 11(1):4504. *Erratum Nat. Commun.* 12:2882. 10.1038/s41467-020-18265-3 32908131 PMC7481196

[B85] Xu B. Lang L. M. Lian S. Guo J. R. Wang J. F. Liu J. (2020). Neuroinflammation induced by secretion of acetylated HMGB1 from activated microglia in hippocampi of mice following chronic cold exposure. *Brain Res.* 1726:146495. 10.1016/j.brainres.2019.146495 31586627

[B86] Yan K. Wang X. Zhu H. Pan H. Wang L. Yang H. (2020). Safflower yellow improves insulin sensitivity in high-fat diet-induced obese mice by promoting peroxisome proliferator-activated receptor-γ2 expression in subcutaneous adipose tissue. *J. Diabetes Investig.* 11 1457–1469. 10.1111/jdi.13285 32356607 PMC7610129

[B87] Yang G. Zhou X. Chen T. Deng Y. Yu D. Pan S. (2015). Hydroxysafflor yellow A inhibits lipopolysaccharide-induced proliferation and migration of vascular smooth muscle cells via Toll-like receptor-4 pathway. *Int. J. Clin. Exp. Med.* 8 5295–5302. 26131104 PMC4483943

[B88] Yang X. W. Li Y. H. Zhang H. Zhao Y. F. Ding Z. B. Yu J. Z. (2016). Safflower yellow regulates microglial polarization and inhibits inflammatory response in LPS-stimulated Bv2 cells. *Int. J. Immunopathol. Pharmacol.* 29 54–64. 10.1177/0394632015617065 26634402 PMC5806736

[B89] Yarar-Fisher C. Bickel C. S. Kelly N. A. Stec M. J. Windham S. T. McLain A. B. (2016). Heightened TWEAK-NF-κB signaling and inflammation-associated fibrosis in paralyzed muscles of men with chronic spinal cord injury. *Am. J. Physiol. Endocrinol. Metab.* 310 E754–E761. 10.1152/ajpendo.00240.2015 26931128 PMC4888537

[B90] Yoshizaki S. Tamaru T. Hara M. Kijima K. Tanaka M. Konno D. J. (2021). Microglial inflammation after chronic spinal cord injury is enhanced by reactive astrocytes via the fibronectin/β1 integrin pathway. *J. Neuroinflammation* 18:12. 10.1186/s12974-020-02059-x 33407620 PMC7789752

[B91] Yu L. Song H. Fang X. Hu Y. (2021). Role of MK2 signaling pathway mediating microglia/macrophages polarization in chronic compression injury of cervical spinal cord. *Ann. Palliat. Med.* 10 1304–1312. 10.21037/apm-20-396 33040559

[B92] Yu S. Zhang H. Hei Y. Yi X. Baskys A. Liu W. (2019). High mobility group box-1 (HMGB1) antagonist BoxA suppresses status epilepticus-induced neuroinflammatory responses associated with Toll-like receptor 2/4 down-regulation in rats. *Brain Res.* 1717 44–51. 10.1016/j.brainres.2019.04.007 30986405

[B93] Zhang H. Duan C. P. Luo X. Feng Z. M. Yang Y. N. Zhang X. (2020). Two new quinochalcone glycosides from the safflower yellow pigments. *J. Asian Nat. Prod. Res.* 22 1130–1137. 10.1080/10286020.2020.1846530 33190510

[B94] Zhang H. Zhi L. Moochhala S. Moore P. K. Bhatia M. (2007). Hydrogen sulfide acts as an inflammatory mediator in cecal ligation and puncture-induced sepsis in mice by upregulating the production of cytokines and chemokines via NF-kappaB. *Am. J. Physiol. Lung Cell Mol. Physiol.* 292 L960–L971. 10.1152/ajplung.00388.2006 17209138

[B95] Zhang Z. Wu Z. Zhu X. Hui X. Pan J. Xu Y. (2014). Hydroxy-safflor yellow A inhibits neuroinflammation mediated by Aβ_1–42_ in BV-2 cells. *Neurosci. Lett.* 562 39–44. 10.1016/j.neulet.2014.01.005 24412680

[B96] Zhou D. Liu B. Xiao X. Dai P. Ma S. Huang W. (2013). The effect of safflower yellow on spinal cord ischemia reperfusion injury in rabbits. *Oxid. Med. Cell Longev.* 2013:692302. 10.1155/2013/692302 24381717 PMC3870863

[B97] Zhou D. Qu Z. Wang H. Su Y. Wang Y. Zhang W. (2018). The effect of hydroxy safflower yellow A on coronary heart disease through Bcl-2/Bax and PPAR-γ. *Exp. Ther. Med.* 15 520–526. 10.3892/etm.2017.5414 29399062 PMC5769294

[B98] Zhu H. Wang X. Pan H. Dai Y. Li N. Wang L. (2016). The mechanism by which safflower yellow decreases body fat mass and improves insulin sensitivity in HFD-induced obese mice. *Front. Pharmacol.* 7:127. 10.3389/fphar.2016.00127 27242533 PMC4876777

